# Safety and efficacy of proactive versus reactive administration of desmopressin in severe symptomatic hyponatremia: a randomized controlled trial

**DOI:** 10.1038/s41598-024-57657-z

**Published:** 2024-03-29

**Authors:** Kamolwan Pakchotanon, Nichanone Kanjanasuphak, Anan Chuasuwan, Pongsathorn Gojaseni, Anutra Chittinandana

**Affiliations:** Division of Nephrology, Department of Internal Medicine, Bhumibol Adulyadej Hospital, Royal Thai Air Force, Bangkok, 10220 Thailand

**Keywords:** Medical research, Nephrology

## Abstract

This randomized controlled trial aimed to evaluate the safety and efficacy of proactive versus reactive desmopressin (DDAVP) strategies in treating severe symptomatic hyponatremia. Conducted from June 20, 2022, to February 20, 2023, it involved 49 patients with serum sodium levels below 125 mmol/L. Patients were assigned to either the proactive group, receiving DDAVP immediately upon diagnosis, or the reactive group, receiving DDAVP only if the serum sodium level tended to be overcorrected. The primary outcome was the incidence of overcorrection. The study revealed no significant difference in the overcorrection incidence between the proactive (16.7%) and reactive (28%) groups (p = 0.54). The change in serum sodium levels at 1, 6, 12, and 24 h were not different, however, at 48 h, the proactive group exhibited a higher but still safe change in serum sodium levels compared to the reactive group (10.3 ± 3.6 mmol/L vs. 7.7 ± 3.6 mmol/L, p = 0.013). Other parameters including time to symptom improvement, total intravenous fluid administered, DDAVP dose, urine volume, hospital stay duration, osmotic demyelination syndrome incidence, and 28-day mortality did not significantly differ between the groups. In conclusion, our findings suggest that there was no significant disparity in overcorrection rates between proactive and reactive DDAVP strategies for treating severe symptomatic hyponatremia. However, further large-scale studies are warranted to validate these results.

## Introduction

Hyponatremia is the most common electrolyte imbalance encountered in clinical practice, affecting up to 13–42% of hospitalized patients^1,2^. It presents with a broad clinical spectrum, ranging from asymptomatic or non-specific symptoms, such as fatigue or lightheadedness, to severe manifestations, including nausea, vomiting, alteration of consciousness, and even coma, which can be life-threatening^3,4^. When serum sodium drops to less than 125 mmol/L, the mortality rate may increase up to 2.1 times^5^. Thus, prompt assessment and treatment of severe hyponatremia is necessary to prevent the development of severe neurological complications, such as seizures and coma, and to reduce the length of hospital stay and prevent further morbidity and mortality. Current treatment guidelines for severe symptomatic chronic hyponatremia recommend empiric intervention with intravenous 3% NaCl to increase serum sodium levels by 4–6 mmol/L within the first 24 h^3,4,6–9^. This measure is considered sufficient for patient stabilization and for preventing further morbidity and mortality. During treatment, close monitoring of clinical, urine volume, and laboratory parameters is recommended to prevent overcorrection, which can lead to osmotic demyelination syndrome (ODS)^10^. Autocorrection of the serum sodium can occur after correction of the underlying causes, such as the administration of saline to patients with volume depletion, the discontinuation of drugs that cause the syndrome of inappropriate antidiuresis (SIAD), and discontinuation of diuretics. These abrupt changes in antidiuretic hormone (ADH) secretion from the posterior pituitary gland can lead to large water diuresis^4,11^.

Desmopressin or 1-deamino 8-D-arginine vasopressin (DDAVP) is a V2 receptor agonist that inhibits water diuresis and controls the increasing rate of serum sodium. Recent studies^12–20^ have shown the clinical benefit of DDAVP in controlling the rate of serum sodium increase when combined with intravenous 3% sodium chloride (NaCl). Studies evaluating DDAVP use for the initial management of hyponatremia have used three different strategies: proactive, reactive, and rescue. Among these, the proactive approach is characterized by the early administration of desmopressin to control renal free water clearance at the beginning of treatment, specifically in patients at increased risk of developing ODS. This strategy, coupled with 3% NaCl, has been demonstrated to effectively elevate serum sodium levels while concurrently reducing the risk of overcorrection^21^. However, most of these studies were retrospective and non-randomized. Thus, further randomized controlled trials are needed to provide a reliable conclusion.

At Bhumibol Adulyadej Hospital, directorate of medical service, Royal Thai Air Force, a tertiary training hospital in Thailand, DDAVP is only used when serum sodium tends to increase beyond the limited range (reactive) or in cases of overcorrection (rescue). Our data collection showed that the incidence of overcorrection was approximately 30% with this standard treatment regimen. Overcorrection may be associated with an increase in hospital stay, mortality rate, and incidence of ODS. Therefore, we conducted this study to compare the safety and efficacy of proactive and reactive DDAVP strategies, in conjunction with 3%NaCl, for treating severe symptomatic hyponatremia.

## Methods

### Study design and participants

This single-center, open-label, randomized controlled trial was conducted at Bhumibol Adulyadej Hospital. The study protocol was approved by the Institutional Review Board of Bhumibol Adulyadej Hospital and was registered in the Thai Clinical Trials Registry (TCTR). This study was conducted in accordance with the ethical principles of the 1964 Declaration of Helsinki and its later amendments. The investigators informed patients or their surrogates concerning the study orally and written informed consent was given before entry into the study.

Eligible participants were adults aged ≥ 18 years with laboratory-confirmed serum sodium < 125 mmol/L and severe symptoms including nausea, vomiting, and changes in consciousness (such as seizures, drowsiness, and coma). Exclusion criteria included hypotension (systolic blood pressure < 90 mmHg and mean arterial pressure < 65 mmHg), anuria (daily urine output < 100 ml), advanced stage chronic kidney disease (glomerular filtration rate estimated by Chronic Kidney Epidemiology Collaboration [CKD-EPI] equation < 30 ml/min/1.73 m^2^), volume overload (e.g., decompensated liver cirrhosis, congestive heart failure), current use of desmopressin, bleeding disorders, pregnancy or breastfeeding, history of the following included cardiac surgery, sustained ventricular tachycardia, ventricular fibrillation, myocardial infarction, traumatic brain injury, increased intracranial pressure within 3 months, and allergic reaction to desmopressin.

### Procedures

The patients were randomly assigned in a 1:1 ratio to receive either proactive or reactive DDAVP strategies in conjunction with 3%NaCl. The allocation sequence used *computer*-generated *random* numbers in a block of fours and allocation concealment. Serum sodium levels were determined through an indirect ion-selective electrode (ISE) method. All patients underwent laboratory tests to assess serum osmolality, blood urea nitrogen (BUN), serum creatinine (Cr), urine osmolality, and urine sodium levels before starting an intravenous drip of 2 ml/kg (maximum 150 ml per dose) of 3%NaCl over 20 min. To ensure the study focused on true hyponatremia, cases of isotonic or hypertonic hyponatremia, such as those induced by hyperglycemia, were excluded. The proactive group received early DDAVP in conjunction with 3%NaCl after the initial serum sodium level was reported and before subsequent serum sodium measurements were taken. The reactive group utilized DDAVP when serum sodium levels tended to increase beyond the limited range. Specifically, DDAVP was administered for a serum sodium rise of 7–8 mmol/L within the first 24 h or 15–16 mmol/L within 48 h, or a 7–8 mmol/L increase from the 24-h to the 48-h period for patients at high risk of ODS—characterized by initial serum sodium < 105 mmol/L, hypokalemia, malnutrition, advanced liver disease, and alcoholism. For those at low risk of ODS—defined as individuals without these high-risk conditions—DDAVP was administered for a serum sodium increase of 9–10 mmol/L within the first 24 h or 17–18 mmol/L within 48 h, or an increase of 7–8 mmol/L from the 24-h to the 48-h period.

**Figure 1:**
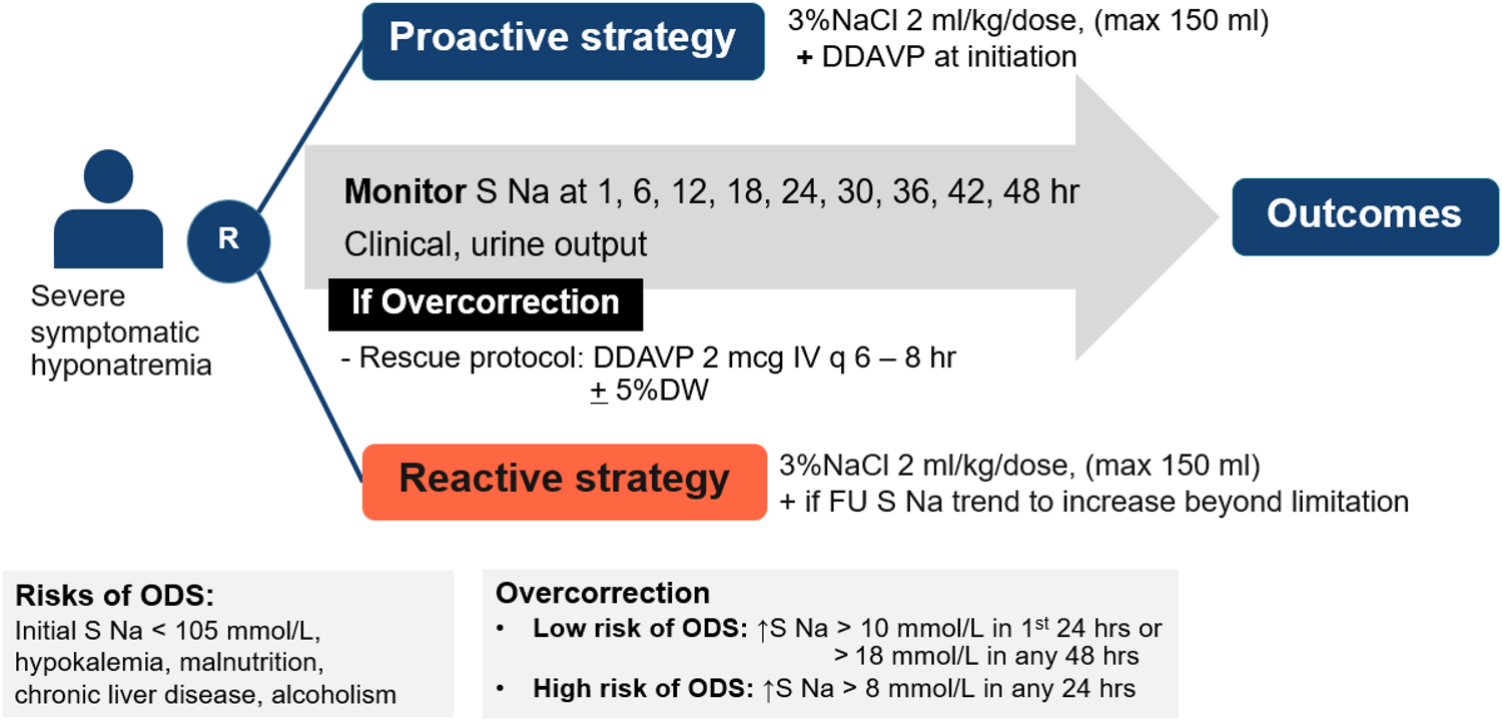
Research protocol. NaCl, sodium chloride; ml/kg, milliliter/kilogram; S Na, serum sodium, DDAVP, Desmopressin or 1-deamino 8-d-arginine vasopressin; DW, dextrose in water; ODS, osmotic demyelination syndrome.

Following the initial differential use of DDAVP, both groups were managed with standard hyponatremia care, involving the investigation and treatment of underlying causes, administration of intravenous hypertonic saline, and administration and repeated doses of intravenous DDAVP at 2 mcg every 6 to 8 h as required. If overcorrection occurred, patients were managed with a rescue strategy. Data collection covered baseline characteristics, clinical data, and serum sodium levels measured at 1, 6, 24, and 48 h post-initial treatment. Although serum sodium was specifically collected at these times, monitoring occurred more frequently at 1, 6, 12, 18, 24, 30, 36, 42, and 48 h. We also recorded urine output, the volume of intravenous fluids administered, and DDAVP dosages. Furthermore, the incidence of ODS and the 28-day mortality rate were assessed. The research protocol is shown in Fig. 1.

### Outcomes

The primary outcome was the incidence of overcorrection between proactive and reactive strategies for the treatment of severe symptomatic hyponatremia. Overcorrection was defined as a serum sodium increase > 10 mmol/L in the first 24 h or > 18 mmol/L in any 48 h in low-risk ODS patients, and > 8 mmol/L in any 24 h in patients at high risk of ODS.

The secondary outcomes included the changes in serum sodium levels at 1, 6, 24, and 48 h after 3%NaCl administration, time to symptom improvement, the total amount of intravenous fluid administered, hospital length of stay, total urine volume, and safety outcomes. The safety outcomes encompassed the percentage of patients requiring relowering treatment, the incidence of ODS—diagnosed through clinical observation of neurological changes, with MRI of the brain performed for confirmation in suspected cases—and the 28-day mortality rate. These measures aimed to compare the safety and efficacy of proactive and reactive strategies in managing severe symptomatic hyponatremia.

### Statistical analysis

Assuming a proportion of participants experiencing overcorrection based on previous literature^13,16,21^, we determined that a sample size of 66 participants would yield 80% statistical power to detect a significant difference in the incidence of overcorrection between the proactive and reactive groups, employing an alpha (ɑ) level of 0.05, beta (ꞵ) of 0.2, and accounting for a 10% dropout rate. For statistical analysis, continuous data were summarized as means ± SD or medians with IQR, contingent on data distribution, and compared using appropriate tests such as the Student t-test or Wilcoxon Rank Sum test. Categorical data were presented as frequencies and percentages and assessed using the Chi-squared test or Fisher’s exact test when appropriate. Results were considered statistically significant when the p-value was ≤ 0.05 for all tests. Data analysis was conducted utilizing R version 4.2.1.

### Ethical approval

The study was approved by the Institutional Review Board of Bhumibol Adulyadej Hospital and was registered in the Thai Clinical Trials Registry (TCTR) with the registration number TCTR20221105001. Informed, written consent was provided by all participants or their surrogates before trial commencement.

## Results

### Patient enrollment and baseline characteristics

**Figure 2:**
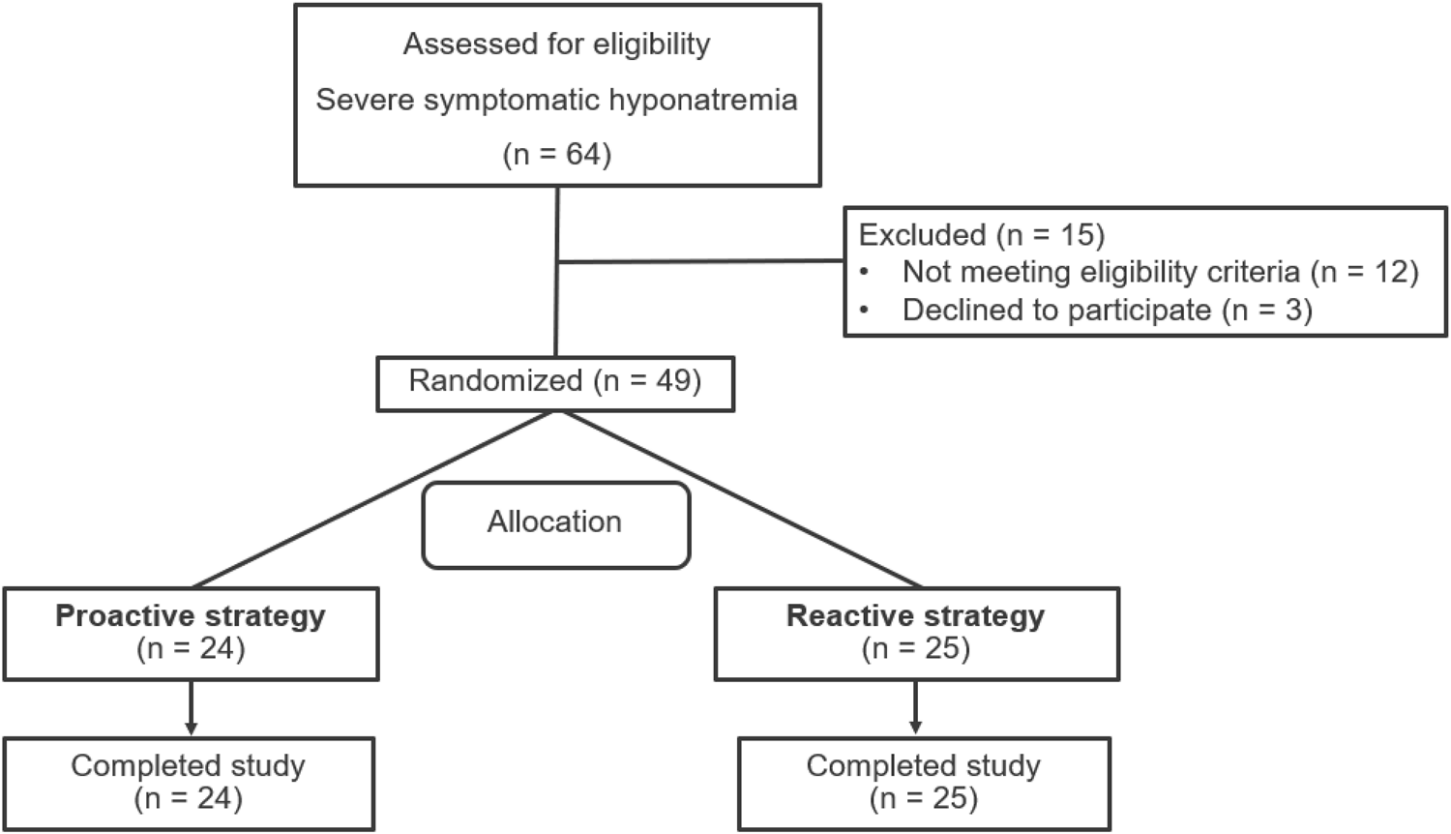
Participant flow diagram.

The study enrolled a total of 49 patients between June 20, 2022, and February 20, 2023, with 24 patients allocated to the proactive group and 25 patients to the reactive group (Fig. 2). The patients had a median age of 72.1 (interquartile range, IQR 67.6, 79.2) years, and 67.3% were women. The mean initial serum sodium level was 115.1 (standard deviation, SD 5.9) mmol/L. The common symptoms at presentation were nausea (55.1%), vomiting (55.1%), and drowsiness (32.7%). Two patients in the reactive group presented with seizures. Twelve (50%) patients in the proactive group and nine (36%) patients in the reactive group were at high risk of ODS. The causes of hyponatremia were SIAD in 23 patients (46.9%), hypovolemia in 18 patients (36.7%), polydipsia in 7 patients (14.3%), and diuretic use in 3 patients (6.1%). Baseline characteristics were no statistical difference between the two groups, as shown in Table 1.

**Table 1:** Baseline characteristics**.**

Characteristics	Proactive strategy **(**n = 24**)**	Reactive strategy **(**n = 25**)**
Demographics		
Age (years), median (IQR)	72.5 (68.8, 76.8)	71.9 (63.1, 79.5)
Male, n (%)	9 (37.5)	7 (28)
BMI (kg/m^2^), mean (SD)	21.1 (2.7)	21.4 (3)
Causes of hyponatremia, n (%)		
SIAD	11 (45.8)	12 (48)
Hypovolemia	9 (37.5)	18 (36)
Polydipsia	3 (12.5)	4 (16)
Diuretic use	3 (12.5)	0 (0)
Co-morbidities, n (%)		
Type2 diabetes mellitus	7 (29.2)	11 (44)
Hypertension	17 (70.8)	17 (68)
Dyslipidemia	11 (45.8)	17 (68)
Psychiatric disease	3 (12.5)	1 (4)
Malignancy	2 (8.3)	2 (8)
History of stroke	2 (8.3)	5 (20)
High risk of ODS, n (%)	12 (50)	9 (36)
Initial lab investigation		
Serum Na (mmol/L), mean (SD)	114.5 (5.7)	115.6 (6.2)
Serum K (mmol/L), mean (SD)	3.8 (0.7)	3.9 (0.8)
Serum Cr (mg/dL), mean (SD)	0.7 (0.2)	0.8 (0.3)
Serum osmolality (mOsm/L), mean (SD)	250.1 (15.3)	250.7 (15.1)
Urine Na (mmol/L), median (IQR)	49.5 (20, 86.2)	44 (20, 63)
Urine osmolality (mOsm/L), mean (SD)	358.7 (159.5)	327.2 (186.2)
Medication		
Diuretic, n (%)		
Hydrochlorothiazide	2 (8.3)	0 (0)
Indapamide	1 (4.2)	1 (4)
Spironolactone	0 (0)	2 (8)
Antidepressant, n (%)	1 (4.2)	2 (8)
Clinical presentation, n (%)		
Nausea	16 (66.7)	11 (44)
Vomiting	17 (70.8)	10 (40)
Drowsiness	6 (25)	10 (40)
Seizure	0 (0)	2 (8)
Coma	0 (0)	0 (0)
Glasgow coma scale score		
Pretreatment, median (IQR)	14 (10, 15)	12 (10, 15)

BMI, body mass index; SIAD, syndrome of inappropriate antidiuresis; ODS, osmotic demyelination syndrome; Na, sodium; K, potassium; Cr, creatinine.

### Primary outcome

The mean of initial serum sodium levels did not differ between the two groups (114.5 [SD 5.7] mmol/L vs. 115.6 [SD 6.2] mmol/L, p = 0.52). The incidence of overcorrection was observed in 4 patients (16.7%) in the proactive group and 7 patients (28%) in the reactive group (p = 0.54), as shown in Table 2.

**Table 2:** Primary and secondary outcomes.

Variables	Proactive strategy (n = 24)	Reactive strategy (n = 25)	p-value
Overcorrection, n (%)	4 (16.7)	7 (28)	0.543
Efficacy, n (%)			
Symptoms improve within 1 h	23 (95.8)	24 (96)	1.000
Glasgow coma scale score			
Pretreatment, median, (IQR)	15 (14, 15)	15 (14, 15)	1.000
Δ sNa (mmol/L), mean (SD)			
Δ sNa at 1 h	3.1 (3.1)	2.7 (2.3)	0.569
Δ sNa at 6 h	2.8 (3)	4 (2.8)	0.136
Δ sNa at 24 h	3.3 (4.1)	5.2 (3.4)	0.096
Δ sNa at 48 h	10.3 (3.6)	7.7 (3.6)	0.013
Time to sNa > 5 mmol/L (hr), median (IQR)	9 (1, 30)	12 (1, 18)	0.862
Time to sNa ≥ 130 mmol/L (day), median (IQR)​	3 (0, 5)	3 (1, 6)	0.715
Length of hospital stay (day), median (IQR)	5 (4, 9.8)	7 (4, 13)	0.416
Volume of fluid administered within 48 h			
NSS (ml), mean (SD)	1805.6 (1185.5)	1660 (1058.8)	0.699
5%DW (ml), mean (SD)	1110 (537)	1565.7 (746.3)	0.315
3%NaCl (ml), mean (SD)	109.17 (25.69)	109.2 (24.14)	1.000
Total DDAVP within 48 h, n(%)			0.127
2 ugm	22 (91.7)	3 (60)	
4 ugm	1 (4.2)	2 (40)	
6 ugm	1 (4.2)	0 (0)	
Urine output (ml), median (IQR)			
Within 24 h	1750 (1500, 2150)	1800 (1400, 2600)	0.904
Within 48 h	3975 (2987.5, 5300)	3400 (2500, 4000	0.085
Safety, n (%)			
Relowering treatment	4 (16.7)	7 (28)	0.543
Incidence of ODS	0 (0)	0 (0)	-
28 day mortality	1 (4.2)	2 (8)	1.000

hr, hour; sNa, serum sodium concentration; Δ sNa, sNa change; NSS, normal saline solution; DW, Dextrose in water; NaCl, sodium chloride; ugm, microgram; ODS, osmotic demyelination syndrome.

### Secondary outcomes

**Figure 3:**
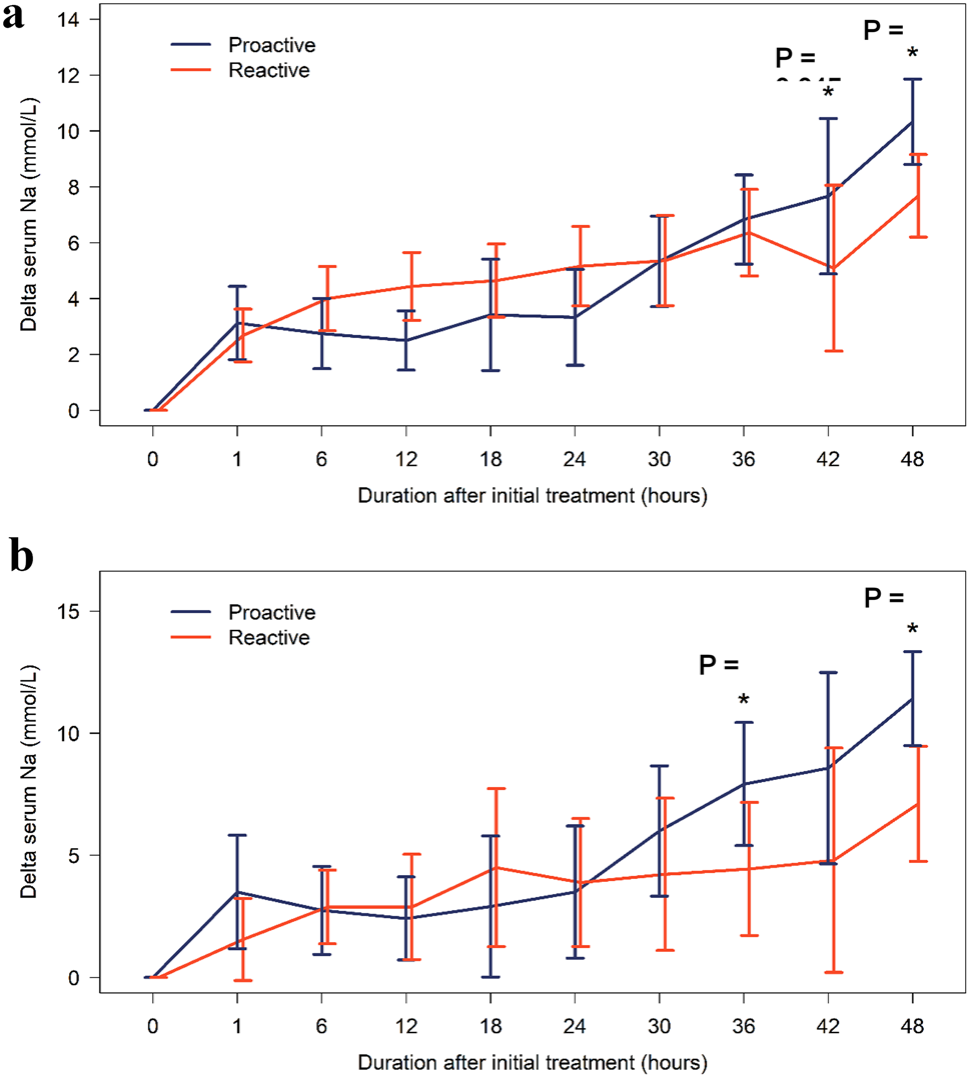
(**a**) Linear mixed-effects model comprising of the trend in the correction of the mean serum sodium concentration over 48 h of treatment between proactive and reactive groups. (**b**) Linear mixed-effects model comparison of the trend in correction of the mean serum sodium concentration over 48 h of high-risk ODS patient’s treatment between proactive and reactive groups (n = 12 in proactive group, n = 9 in reactive group).

There was no significant difference in the total amount of normal saline solution and 5% dextrose water administered between the two groups. The average dose of DDAVP administered per patient within 48 h was 2.25 mcg in the proactive group and 2.8 mcg in the reactive group. However, this difference was not found to be statistically significant (p = 0.100). The changes in serum sodium levels at 1, 6, and 24 h were not significantly different between the two groups. However, at 48 h, the proactive group showed a greater increase in serum sodium levels compared to the reactive group (10.3 [SD 3.6] mmol/L vs. 7.7 [SD 3.6] mmol/L; p = 0.013, as shown in Table 2). The trend in the correction of mean serum sodium concentration over 48 h of treatment between the overall proactive and reactive groups and the high-risk ODS subgroup was shown in Fig. 3a and b, respectively. These figures revealed that the proactive group had a greater increase in mean serum sodium levels than the reactive group at 48 h after initial treatment. However, these increments remained within the safety limits. No significant difference was observed between the groups in terms of time to symptom improvement, time to an increase in serum sodium of 5 mmol/L, or length of hospital stay. No events of ODS occurred in either group. One patient in the proactive and two patients in the reactive group died with no death attributed to complications of hyponatremia.

## Discussion

Our study aimed to compare the incidence of overcorrection between proactive and reactive DDAVP strategies for the treatment of severe symptomatic hyponatremia, stratified according to ODS risk. There was no significant difference in the incidence of overcorrection between the two groups.

The current recommended therapeutic approach^3,4,6,7,9^ for managing severe symptomatic chronic hyponatremia emphasizes timely intervention with intravenous hypertonic saline to achieve a targeted rise in serum sodium levels, aiming for an increment of 4–6 mmol/L within the first 24 h. The guidelines stipulate a maximum increase of no more than 10 mmol/L in the initial 24 h or 18 mmol/L across any 48-h interval for patients with chronic hyponatremia at low risk of osmotic demyelination syndrome (ODS). For those at high risk, the serum sodium should not increase by more than 8 mmol/L within 24 h. While previous studies have demonstrated that DDAVP can mitigate the risk of overcorrection^21^, some instances have shown it leading to inadequate sodium level increases or even decreases^16,19,20^, especially with unrestricted fluid intake^11^.

Notably, our study reported a lower incidence of overcorrection compared to previous literature^21^. Frequent monitoring of clinical and laboratory parameters, along with the prompt adjustment of management in both groups, may have contributed to this lower incidence of overcorrection and the absence of statistically significant results. Frequent monitoring and prompt treatment adjustments may be the key to preventing overcorrection. Moreover, a prior study suggested that a urine volume exceeding 250 mL/hour could lead to a rapid increase in serum sodium levels^17^. In our study, the median urine volume during the initial hour after treatment remained below this threshold for both groups, resulting in a minor increase in serum sodium levels. However, this was sufficient to alleviate hyponatremia symptoms.

At 48 h following the initial treatment, the proactive DDAVP group demonstrated a higher increase in mean serum sodium levels compared to the reactive group. This distinction suggests that early DDAVP administration in the proactive group might have facilitated controlled sodium correction once the underlying causes of hyponatremia were addressed, often eliminating the need for further doses on the second day, contrasting with the reactive approach where DDAVP administration was based on evolving sodium levels, potentially leading to later interventions. However, this increase remained within the safe range, and there were no significant differences in the amount of 3%NaCl, NSS, or 5%DW administered between the two groups within 48 h. Our study also found no differences in prolonged 3% NaCl infusion times, total fluid amount, or length of stay in severe hyponatremia between the proactive and reactive DDAVP strategies, which contrasts with previous literature^19^.

As recognized, overcorrection in hyponatremia management depends on crucial factors, including the rapid sodium level increase post-treatment and patients' susceptibility to serum sodium rise due to autocorrection, like ADH suppression after volume repletion in hypovolemic situations, causing significant diuresis. Additionally, the risk of polyuria escalates in patients with low initial urine osmolarities or inadequate prior solute intake, necessitating meticulous solute and fluid management. Both proactive and reactive DDAVP administration strategies may be suitable for preventing overcorrection. However, for patients with severe symptomatic hyponatremia who are particularly prone to overcorrection—especially those at high risk for ODS where consistent monitoring of laboratory values and urine output is challenging—early use of DDAVP can be considered a proactive and viable strategy.

In our study, we did not utilize predictive sodium change equations like the Voets formula^22^, which is tailored for DDAVP clamping where urine osmolality is consistently fixed as in SIAD conditions. The decision was influenced by the varied causes of hyponatremia in our population and the hospital's constraint in promptly providing urine osmolality readings. Consequently, our approach emphasized clinical judgment and consistent serum sodium monitoring. It is noteworthy that initial urine osmolarity findings, despite not being immediate, indicated no significant differences between the groups.

A notable strength of our study is its status as the first randomized controlled trial assessing proactive and reactive DDAVP strategies for treating severe symptomatic hyponatremia, with overcorrection categorized based on ODS risk. Despite excellent protocol adherence, limitations include a small sample size potentially affecting power, confinement to a single center potentially limiting generalizability, and limited documentation of oral fluid intake due to emergency department management. Additionally, while urine osmolality results were not used in real-time for decision-making due to laboratory constraints, urine specific gravity was used for immediate clinical assessments, albeit with limited precision for predicting serum sodium changes.

## Conclusions

Our study found no significant difference in the incidence of overcorrection between proactive and reactive DDAVP strategies, in conjunction with 3%NaCl, for treating severe symptomatic hyponatremia. Frequent monitoring and prompt adjustment of treatment may be crucial in preventing overcorrection. While the proactive approach shows promise, especially where continuous monitoring is challenging, definitive conclusions require further large studies.

## Data Availability

Data generated from this study is not publicly available due to patient privacy concerns. Participants consented to the study but did not consent to the public sharing of their data. Requests for data that underpin the findings reported in this article may be made to the corresponding author, subject to adherence to participant confidentiality and ethical considerations.
